# Bilateral theta-burst magnetic stimulation influence on event-related brain potentials

**DOI:** 10.1371/journal.pone.0190693

**Published:** 2018-01-05

**Authors:** Nuno Pinto, Marta Duarte, Helena Gonçalves, Ricardo Silva, Jorge Gama, Maria Vaz Pato

**Affiliations:** 1 CICS-Health Sciences Research Centre, Faculty of Health Sciences, University of Beira Interior, Covilhã, Portugal; 2 Dr. Lopes Dias School of Health - Polytechnic Institute of Castelo Branco, Castelo Branco, Portugal; 3 Department of Mathematics - University of Beira Interior, Covilhã, Portugal; 4 Sousa Martins Hospital, Guarda Local Health Unit, Guarda, Portugal; Shanghai Mental Health Center, CHINA

## Abstract

Theta-burst stimulation (TBS) can be a non-invasive technique to modulate cognitive functions, with promising therapeutic potential, but with some contradictory results. Event related potentials are used as a marker of brain deterioration and can be used to evaluate TBS-related cognitive performance, but its use remains scant. This study aimed to study bilateral inhibitory and excitatory TBS effects upon neurocognitive performance of young healthy volunteers, using the auditory P300’ results. Using a double-blind sham-controlled study, 51 healthy volunteers were randomly assigned to five different groups, two submitted to either excitatory (iTBS) or inhibitory (cTBS) stimulation over the left dorsolateral pre-frontal cortex (DLPFC), two other actively stimulated the right DLPFC and finally a sham stimulation group. An oddball based auditory P300 was performed just before a single session of iTBS, cTBS or sham stimulation and repeated immediately after. P300 mean latency comparison between the pre- and post-TBS stimulation stages revealed significantly faster post stimulation latencies only when iTBS was performed on the left hemisphere (p = 0.003). Right and left hemisphere cTBS significantly delayed P300 latency (right p = 0.026; left p = 0.000). Multiple comparisons for N200 showed slower latencies after iTBS over the right hemisphere. No significant difference was found in amplitude variation. TBS appears to effectively influence neural networking involved in P300 formation, but effects seem distinct for iTBS vs cTBS and for the right or the left hemisphere. P300 evoked potentials can be an effective and practical tool to evaluate transcranial magnetic stimulation related outcomes.

## Introduction

Transcranial magnetic stimulation (TMS) has become an essential tool for manipulation of cortical activity, thereby allowing the study of the functional organization of the human brain [1]. The continual development of techniques such as repetitive TMS (rTMS) and patterned rTMS, enhances their potential as a tool for clinical treatment of several psychiatric and neurological diseases [2–6]. TMS has been shown as a safe approach to non-invasive research of cognitive functions, both in healthy and pathologic brain. However, research focusing upon the cognitive therapeutic potential of rTMS over the last years has shown contradictory results, thereby perpetuating some doubts over its mechanisms [7, 8].

It is known that stimulus characteristics such as frequency, intensity, train length or total number of pulses can induce lasting inhibitory or excitatory after-effects [4]. Theta-burst stimulation (TBS) is a form of patterned rTMS which has some advantages including lower stimulation intensity, a short stimulation period and a more prolonged after-effect as compared to other rTMS protocols, both the excitatory (iTBS) and the inhibitory (cTBS) forms [9], and is additionally regarded by some authors to be safer than traditional rTMS [4, 10].

Event related potentials (ERPs) are cerebral responses to external stimuli, which reflect the neurophysiology of cognition [11, 12] and may be used to study the cognitive effects of TBS. The auditory P300, directly dependent upon subject’s attention and discrimination, is the most extensively researched ERP component, resulting from the discrimination of rare, task-relevant stimuli, generally using an oddball paradigm. Predominantly reflecting processing speed, is an important tool in the study of cognitive processes and memory in normal subjects and in psychopathology, as its delay can be used as a marker of cognitive deterioration [13, 14]. Playing a less prominent role in ERP studies, the N200 potential also yields important information regarding cognitive evaluation, as it represents the initial, subconscious processing of the stimulus involved in the oddball task, leaving the translation of more advanced and purposeful stages of task processing to P300.

Thus far, the use of ERPs remains scant [7, 8], and there is still little research on auditory P300 and TBS. Therefore, in order to study TBS effects upon neurocognitive performance using a ERP evaluation tool, we delineated a study combining auditory P300 and TBS applied to young healthy volunteers. Our objectives were: a) to study the effects of a single TBS (iTBS or cTBS) session upon auditory P300 performance, b) to analyse whether the stimulated side originates any lateralization on parietal P300 responses and c) to evaluate whether TBS protocol has any influence upon the volunteers’ reaction time during P300 testing.

## Materials and methods

### Subjects and study design

This was a double-blind sham-controlled study, involving healthy volunteers that were recruited after general advertisement with medical students enrolled at the Faculty of Health Sciences, University of Beira Interior, Covilhã, Portugal. Students were selected if they were between 18 and 30 years-old, and after answering a confidential screening questionnaire. Exclusion criteria included being left-handed or ambidexter; previous brain injury and/or severe head trauma; epilepsy or history of convulsions; presence of major medical illness (including neuropsychiatric diseases), intake of any medication during testing, pregnancy, implanted devices or foreign metal articles, sleep deprivation, alcoholism and history of drug intake [4]. All volunteers were instructed to avoid sleep deprivation, alcoholic beverages or other toxic/stimulant substances 24 hours prior to the application of the technique.

Volunteers were then randomly assigned to five different groups: two groups with active stimulation to the left dorsolateral pre-frontal cortex (DLPFC)—Group A (iTBS) and Group B (cTBS), two other groups with active stimulation over the right DLPFC (Group D (iTBS) and Group E (cTBS) and finally, a placebo group—Group C (Sham).

After complete explanation of the procedures, all subjects signed a written informed consent. The study was approved by the Faculty of Health Sciences UBI Ethics Committee (no. CE-FCS-2011-001), in conformity with the Declaration of Helsinki.

### Theta burst stimulation (TBS)

TBS was performed under medical supervision at FCS-UBI facilities, using a 70 mm figure-8 coil with a MagVenture MagPro^®^ G3 X100 5.0.1 and recording EMG activity in a Dantec^™^Keypoint^®^—Keypoint.net v.2.03. Stimulation comprised a biphasic pulse waveform and antero-posterior (A-P) current direction in single pulse, iTBS and cTBS [4].

Stimulation intensity was defined using the active motor threshold (AMT), which consisted of the minimal stimulation intensity over the motor cortex that was necessary to produce a 150–200μV amplitude motor evoked potential (MEP) of the contralateral *abductor pollicis brevis* (APB), on more than five out of ten trials, while maintaining a voluntary mild contraction, using visual feedback. Active stimulation was performed over the right or left DLPFC area that can be defined as 5 cm rostral of the region from which the most prominent motor response of the contralateral APB muscle can be recorded [8, 9, 15].

The TBS protocol consisted of bursts of 3 pulses delivered at 50 Hz every 200 ms (i.e. at 5Hz), at an intensity set to 80% AMT [11]. In the cTBS protocol the bursts were delivered without interruption, up to a total of 600 pulses. iTBS also comprised 600 pulses, but the bursts were delivered at 5 Hz during 2 s (groups of 10 bursts), repeated every 10 seconds [9].

Sham stimulation used the same coil, tilted away from the scalp at a 90 degree angle, but maintaining contact and sound (intensity reduced to 50% AMT), thereby giving the impression that the subject was being stimulated, although this stimulus does not reach cortical neurons [4, 8]. During protocol application, subjects were seated in a comfortable declinable armchair and were told to relax and avoid any head movements.

### P300

Auditory P300 recording was carried out in a quiet room, using an 8 channel Keypoint.net v.2.03. Active electrodes were placed in Cz, Pz, P3 and P4 of the 10/20 international system, with an anterior reference, trying to achieve a more accurate lateralization of the waves recorded in the right and left parietal electrodes. All recording sites were cleaned with alcohol and abraded to maintain a resistance below 5 kΩ. [11, 16, 17]. A time constant of 1 second was used together with a high frequency filter of 50 Hz, with a time base of 1000 ms, using an automatic overload rejection mode. The auditory oddball paradigm consisted of 80% frequent stimuli presentation, 1000 Hz and 50 ms of duration, randomly mixed with a 20% target stimulus, 2000 Hz and 100 ms of duration. Both used a minimal intensity of 65 dB HL. Stimuli were presented binaurally, with a random interval between 1 and 2 seconds. Each complete study recorded at least 400 stimuli (minimum of 100 target), divided into two series, and subjects were instructed to remain calm and relaxed, avoid blinking and to concentrate upon a focus point. Subjects were then asked to press a button for the rare stimuli as quickly as possible with the dominant hand in order to ensure attention and collaboration [11, 18]. The chosen parameters were measured from the mean waveform of the two reproducible series and the epochs for the target and non-target tones were analysed separately. The largest negative peak, occurring between 160–260 ms, was considered as the N200. The P300 was defined as the largest positive peak arising after the N1, P2 and N2 components, increasing in amplitude at the posterior areas and occurring between 220–600 ms. Amplitude was measured in the N2-P3 complex, between the maximum negativity and positivity components [11, 12, 19, 20].

### Experimental design

The study design comprised three different timepoints for assessment, labelled as pre-TBS, TBS stimulation and post-TBS. Stimulation was always performed at the same time of day and randomly assigned to each volunteer according to the respective group. Each subject was submitted to a single TBS session on the DLPFC. The order of real and sham sessions was also randomized and counterbalanced across subjects. Only one member of the investigation team was aware of the type of stimulation applied. In pre-TBS stage, baseline P300 recording was performed. This step was followed by all the procedures regarding TBS protocol, performing either iTBS, cTBS or sham stimulation. Immediately after TBS or sham stimulation, the second auditory P300 recording was performed (post-TBS). Protocol available at: dx.doi.org/10.17504/protocols.io.kr3cv8n

### Statistical analysis

Chi-square and Levene tests were used to study if there were any significant differences between groups. Normality was evaluated using Kolmogorov-Smirnov and Shapiro-Wilk tests. Due to the relative small number of group elements and data characteristics, we needed a robust nonparametric analysis test to evaluate pre-post stimulation mean result comparisons and multiple group comparison test, thus we used the R software package: Nonparametric Analysis of Longitudinal Data in Factorial Experiments (nparLD) [21]. Analyses were performed using IBM SPSS Statistics 20^®^ and R version 3.0.0., and the significance level was p < 0.05.

## Results

### Volunteers

This study involved 51 healthy volunteers (31 female and 20 male, aged 19–30 years, mean = 22.84 +/- 1.98), and all study groups (Group A n = 10; Group B n = 10; Group D n = 10; Group E n = 11, and Group C n = 10), were matched in terms of age and gender.

### Pre-stimulation—N200 and P300

For all groups, N200 mean latency pre-stimulation ranged between 176.98 +/- 30.21 ms over Pz and 181.73+/- 23.05 ms over Cz. As for P300, the lowest mean latency was obtained over Cz– 255.65 +/- 45.07 ms—and the highest over P3—259.57+/-54.81 ms. Overall maximum latency recorded reached 256 ms and 483 ms, for N200 and P300 respectively. Amplitudes recorded regarding N2-P3 difference, showed mean results between 4.72 +/- 3.12 μV over Cz and 5.10 +/- 3.85 μV over Pz, with a maximum amplitude of 19.9 μV. Signalizing the rare stimuli by pressing the button on our oddball paradigm achieved an overall reaction time mean of 316,24 +/- 57,04 ms, ranging from 217 to 468 ms.

### Pre- and post-stimulation latencies

**Fig 1:**
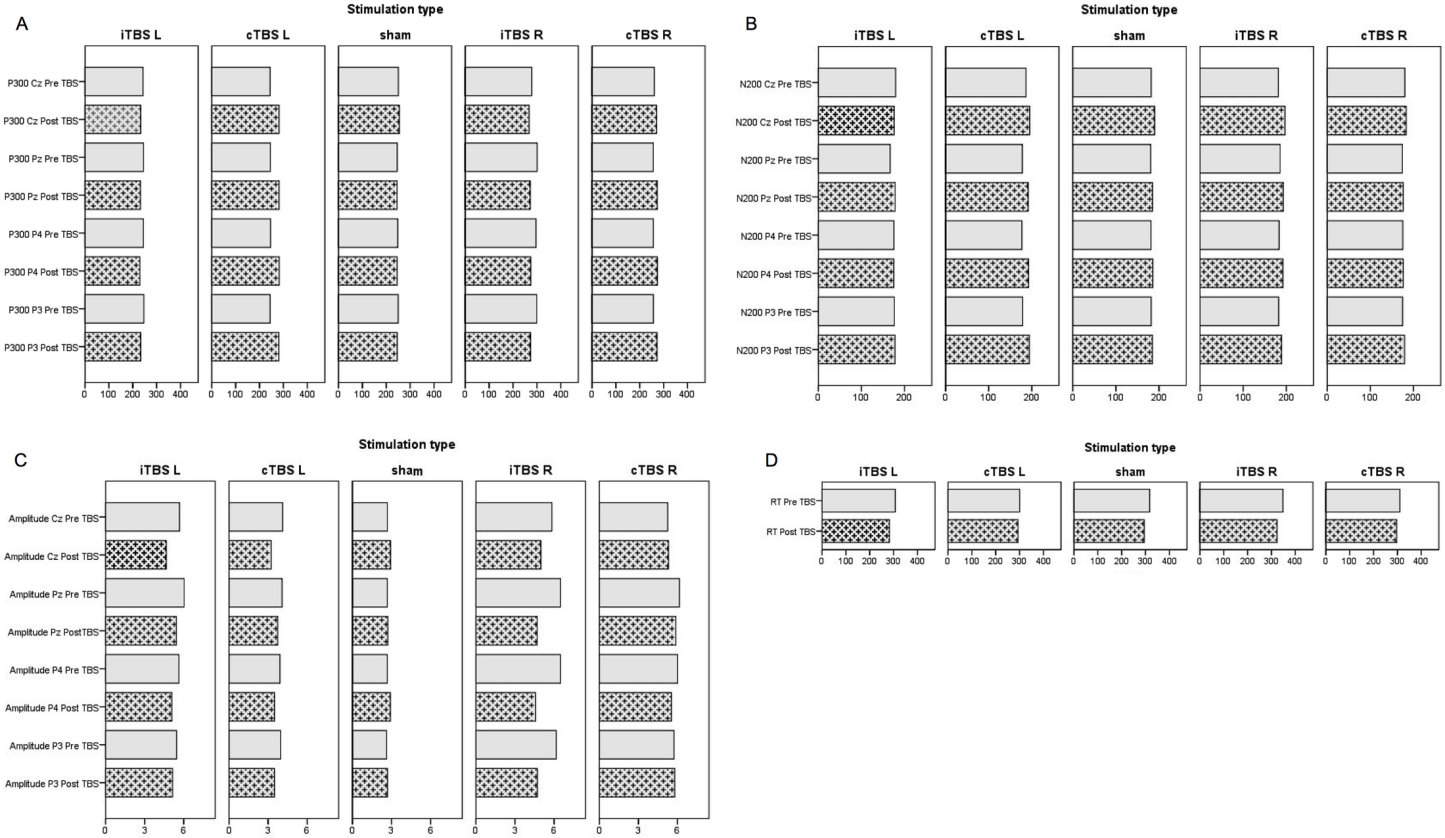
ERP results per stimulation group. P300 latency (A), N200 latency (B), Amplitude (C) and Reaction Time (D).

Pre-stimulation and post-stimulation latencies, amplitudes and reaction times distributed per stimulation group are shown in Fig 1.

Comparison of P300 latencies between the pre- and post-TBS stimulation stages are shown in Table 1.

**Table 1:** Group comparison—Pre vs Post stimulation—P300 and N200 latencies.

	iTBS L		cTBS L		Sham		iTBS R		cTBS R	
	Mean Dif. (ms)	p-value^a^	Mean Dif. (ms)	p-value^a^	Mean Dif. (ms)	p-value^a^	Mean Dif. (ms)	p-value^a^	Mean Dif. (ms)	p-value^a^
P300 Cz Pre	-9,7	0.095	38,4	**0.009**	3,8	0.506	-10,8	0.604	9,91	*0*.*062*
P300 Cz Post										
P300 Pz pre	-12,9	**0,003**	36,8	**0,000**	-0,8	0,822	-28,4	0.084	16,64	**0.026**
P300 Pz Post										
P300 P4 Pre	-14,2	**0.006**	36,4	**0.000**	-2,4	0.829	-21,4	0.829	16,55	**0.009**
P300 P4 Post										
P300 P3 Pre	-13,3	**0.005**	37	**0.001**	-3,7	0.515	-26,2	0.345	15,18	**0.035**
P300 P3 Post										
N200 Cz Pre	-3,4	0,149	8,8	0,960	7,9	0.238	15,5	**0.006**	3,55	0.709
N200 Cz Post										
N200 Pz Pre	11,6	0.411	13,6	0.277	4,3	0.398	7,3	0.449	1,73	0.837
N200 Pz Post										
Reaction Time Pre	-24,2	**0,000**	-6,1	0,629	-22,4	**0,025**	-24,1	0,052	-13,45	0,176
Reaction Time Post										

^a^nonparametric—nparLD pakage

Differences were detected between groups, in terms of stimulation characteristics. iTBS groups showed a tendency towards decreasing P300 latencies after stimulation and cTBS groups showed a tendency towards a slower response time. In contrast, the sham group did not show a clear tendency.

Sham and right hemisphere iTBS groups showed no significant differences between the pre and post evaluations (nonparametric—nparLD package). iTBS over the left hemisphere showed significantly faster post stimulation latencies, mainly over the parietal recording sites (p = 0.003, p = 0.006 and p = 0.005 for Pz, P4 and P3, respectively). cTBS over the left hemisphere significantly influenced P300 latency over all recording topographies, causing a delay in the P300 wave. In the right hemisphere, cTBS stimulation was associated with a significant parietal ERP delay (p = 0.026, p = 0.009 and p = 0.035 for Pz, P4 and P3, respectively).

In terms of N200, latency showed a significant difference only when iTBS was performed on the right hemisphere. Contrasting with P300 behaviour to excitatory stimulation, N200 displayed longer latencies after stimulation. The remaining groups showed relatively small and inconstant changes in mean latencies.

### Pre- and post-stimulation reaction times

Comparison of reaction times between the pre- and post-TBS stimulation stages are shown in Table 1.

All groups showed faster reaction times in the second ERP evaluation, after TBS and sham stimulation, but this was only significant in the sham group (mean difference = -22.4 ms; p = 0.000) and the left iTBS group (mean difference = -24.2 ms; p = 0.025). In contrast, right iTBS group only showed a trend towards reaction times being significantly faster (mean difference = -24.1 ms; p = 0,052).

### Pre- and post-stimulation amplitudes

Comparison of ERP amplitudes between the pre- and post-TBS stimulation stages are shown in Table 2.

**Table 2:** Group comparison—Pre vs Post stimulation—ERP amplitude.

	iTBS L		cTBS L		Sham		iTBS R		cTBS R	
	Mean Dif. (μV)	p-value^a^	Mean Dif. (μV)	p-value^a^	Mean Dif. (μV)	p-value^a^	Mean Dif. (μV)	p-value^a^	Mean Dif. (μV)	p-value^a^
N2P3 Cz Pre	-1,01	0.189	-0,89	0.582	0,24	0.543	-0,84	0.295	0,06	0.876
N2P3 Cz Post										
N2P3 Pz Pre	-0,6	0.980	-0,33	0.850	0,04	0.963	-1,78	0.944	-0,28	0.454
N2P3 Pz Post										

^a^nonparametric—nparLD pakage

ERP amplitudes before and after stimulation in all groups, except for the sham group showed a trend towards a slight decrease after TBS, but no significant difference was found.

### Group comparison—Stimulation vs Sham—P300

Comparison of Pz P300 results across all stimulation groups is shown in Table 3.

**Table 3:** Stimulation group vs Sham group multiple comparison test—P300 & N200 latencies.

	P300 Lat. Pz	P300 Lat. Cz	N200 Lat. Pz	N200 Lat. Cz
	p-value^a^	p-value^a^	p-value^a^	p-value^a^
iTBS L vs Sham	**0.024**	0.805	0.250	0.764
cTBS L vs Sham	**0.001**	**0.016**	0.201	0.317
Sham vs iTBS R	0.167	0.837	0.262	**0.024**
Sham vs cTBS R	**0.042**	0.082	0.414	0.280

^a^nonparametric ANOVA nparLD

When we evaluate the outcomes through a multiple comparisons test, P300 latency over Pz results showed significant differences between the sham group and the left iTBS group (p = 0.024), sham and left cTBS goups (p = 0.001) and finally between sham and right cTBS groups (p = 0.042).

Comparing groups using Cz P300 (Table 3), the only significant difference occurred between the sham and the left cTBS groups (p = 0.016), with much slower latencies recorded after actual cTBS stimulation.

### Group comparison—Stimulation vs Sham—N200

Multiple comparisons for N200 (Table 3) showed no significant differences over Pz recordings. N200 behaviour over Cz was significantly different between sham and right iTBS groups, in this case because N200 was slower after excitatory TBS over the right hemisphere. ERP behaviour over P3 and P4 followed overall Pz results after pre- and post-stimulation, not showing any significant lateralization.

## Discussion

The main goal of our work was to evaluate human cortical and subcortical network dynamics to TBS, via electrophysiological assessment using the auditory P300 ERP. Introducing a sham controlled design trial, we tried to verify if the effects were distinct for iTBS vs cTBS and for the right or the left hemisphere. To our knowledge, this is the first study that compared both excitatory and inhibitory TBS over the right and left DLPFC, evaluating its effects using neurophysiological tests like the auditory P300, with a placebo control group, in a young adult healthy population. Our sham-controlled results showed that ERPs responded differently to stimulation type and lateralization. Significantly slower P300 latencies were recorded over parietal locations after left and right inhibitory stimulation but faster P300 latencies were found only after excitatory stimulation over the left DLPFC. No apparent latency lateralization was found as P300 over P3 and P4 followed the same outcomes as the P300 recorded over Pz. Amplitudes showed no significant variation after cTBS or iTBS in either hemispheres. Reaction times behaved differently also with faster reaction times in the excitatory and sham groups, but with no significant changes in the inhibitory groups.

Using both inhibitory and excitatory TBS protocols, we found that the parietal P300 showed significantly slower latencies after cTBS stimulation bilaterally but the parietal P300 responses were significantly faster only after iTBS over the left cortex. These results suggest that the inhibitory protocol is capable of a more intense or more effective interference over the cerebral circuits that are implicated in P300 formation than excitatory TBS, as it seems to be able to modulate both hemispheres. Supporting these findings, Kaller et al. found interesting results when testing hemispheric relevance using bi-hemispheric cTBS and the Tower of London task. Their results showed that initial planning times could be influenced differently either by stimulating the right or the left hemisphere, with results directly dependent of hemisphere dominance—right hemisphere inhibition resulted in increased planning times and contralateral inhibition showed faster planning [22]. Such evidence is similarly defendable for ERPs global performance, since using a inhibitory stimulation over the frontal area originated decreases ERP amplitude in a modified P300 protocol [23].

Our results also propose an asymmetrical response to excitatory stimulation, since iTBS in our study seemed to be more effective over the left hemisphere, and P300 showed significantly slower latencies over Cz only after left cTBS. Leftward susceptibility to be more easily modulated was detected in other studies with excitatory stimulation, as shown by the faster latencies found after high frequency rTMS over the left hemisphere [24]. Overall, right hemisphere stimulation results tend to reveal fewer changes in ERP parameters, as showed when administering inhibitory rTMS over the right DLPFC [25, 26], or excitatory rTMS over the right DLPFC [24]. Although asymmetries are reported, our overall recordings of P300 over the left and right parietal areas showed the same results as the P300 recorded over Pz. These findings suggest that lateralized cTBS and iTBS can influence the initial P300 neuronal generator behaviour but not the following bilateral wave formation and spreading. Our findings can be associated to TBS/rTMS modulation capacity to influence neurotransmitter production, as neurotransmitters trigger intracortical excitatory and inhibitory postsynaptic potentials that are the base for ERP formation. Magnetic stimulation capacity to modulate neurotransmitter dopaminergic and glutamatergic connection is known, especially if applied to the prefrontal cortex, and these neurotransmitter assume utmost importance in P300 formation [27, 28]. Previous studies showed that high frequency magnetic stimulation increases anterior brain glutamate levels, in some cases with a left lateralization [29–32]. It is also known that dopamine modulation can influence both task performance testing and also event related potentials [33, 34]. ERP latencies and amplitudes can be influenced by dopaminergic function, impacting cognitive speed processing and also neural resources magnitude allocation to a specific task. Magnetic stimulation can similarly impact dopaminergic function, with some studies showing that high frequency stimulation administered to left prefrontal cortex increases dopamine release [35, 36]. Research also showed that in some studies this effect had also some degree of lateralization, as only the left hemisphere stimulation resulted in either dopamine increase after excitatory stimulation or impaired dopamine release after inhibitory stimulation [33–40]. These findings can strongly be correlated with our P300 latency results, since it is likely that cTBS over bilateral DLPFC can have a direct negative impact in either or both glutamate and dopamine production, essential in the electrogenesis of P300 potentials, resulting in ERP delay, even though it may be predominant over the ipsilateral hemisphere. We also found asymmetrical results, as it appears to exist a superior TBS influence over the left DLPFC, especially effective for iTBS and these findings can be related to the reported apparent iTBS superior capability to influence left hemisphere glutamatergic and dopaminergic release. Assuming that P300 test performance is related to mental processing speed affected by attentional processing and cognitive operations, as shown in previous works [41], we can also assume that iTBS over the DLPFC worked has a facilitator of the cognitive and executive process.

As for N200 performance, reflecting the initial subconscious process of the ERP oddball task, our results showed small variations across the groups, except for the right iTBS group, revealing significantly slower N200 latencies, apparently divergent to P300 behaviour to excitatory stimulation. Previous experimental studies pointed to a left hemisphere N200 dominance, predominantly over the anterior mid-cingulate cortex, evaluated by magnetic resonance images, suggesting also a functional and neuroanatomical dissociation between N200 and P300 potentials [42]. We believe that this anatomical dissociation may explain the different P300 vs N200 response to TBS. In this case, the right inter-hemispheric inhibitory connectivity capabilities could have been potentiated by the right-sided iTBS [43–45], thus negatively influencing the N200 dominant left hemisphere, unbalancing right-left basal equilibrium, resulting in poorer N200 performance. Since N200 reflects the initial ERP phase, this result can also be related to right iTBS poorer P300 performance discussed earlier.

It is known that P300 amplitude is associated to the amount of attentional neuronal resources allocated throughout the P300 task, but amplitude evaluation is not straightforward, as it implies a relationship between attention and working memory that can originate higher amplitudes for easy targets and lower amplitude for more complex tasks, requiring more memory load [46, 47]. In our groups, even though the task was not complex, probably our baseline psychological conditions were not ideal, as we were introducing a new, and somewhat unknown stimulation technic to our volunteers, that could have induced some anxiety. Our results did not reveal any significant change in ERP amplitude, neither in the stimulated groups or in the sham group. Our lack of significant changes in P300 amplitude, associated to a low baseline amplitude P300, could be related to a state of low excitability or a limited capacity to better allocate attentional neuronal resources, possibly related to the TMS protocol-disturbing physiological volunteer estate. It is also well established that P300 activity is influenced by individual internal physiologic state, ranging from circadiam rhythms to fatigue and physical state [48]. Base line ERP results revealed latency and amplitude characteristics that can be explained by factors like our sample of young university students, capable of promoting a lower latency baseline ERP, and technical aspects as reference electrode position, as it is argued that anterior references are positioned within brain’s electrical fields of the auditory ERP, being capable of voltage gradients which vary across subjects [16, 41]. So, even though our primary aim was to reduce possible amplitude asymmetry by electrode location and impedance discrepancies, this fact could have influenced amplitude and even latency baseline results [49].

When evaluating reaction time in ERP task we must remember that TMS has the capacity to induce local, trans-synaptic and system-level effects. We know as well that this ERP protocol involves a motor response and apparent significant involvement of the anterior cingulate cortex [48]. The fact that all groups, including sham group, tended to shorter reaction times suggests a mere habituation process. But careful analysis shows that stimulation type may influence this process because of right and left cTBS groups response speed wasn’t significantly as fast as their counterparts. This result suggests that cTBS inhibitory capacity negatively influenced bilateral cerebral networking, preventing these groups to perform as fast as they normally would, supporting the notion that even though the DLPFC could be the most active region, it can activate cortical network relays, including deep subcortical relays, thus influencing motor response processes [35].

Using the TBS-P300 combination appears to be a useful approach to monitor stimulation effects, especially if applied when evaluating neurologic and psychiatric diseases, either in rehabilitation or diagnosis. This method may be also important to better understand neural network processing as it allows studying the direct and indirect influence of specific cortical and subcortical connectivity over cognitive performance. As mentioned, previous studies combining rTMS and event related potentials, magnetic stimulation tends to modulate brain responses accompanying the excitatory or inhibitory effects associated with high or low frequency stimulation, respectably, but most studies used only one stimulation type and one stimulation site, mostly without placebo control. Knowing that some previous results were even negative using bilateral inhibitory stimulation [50], a broader study using iTBS and cTBS was clearly necessary. Regardless the fact that there were already studies evaluating the effect of rTMS on the human cortex and the capacity to impact scalp ERPs, the significant variability in application technics and in some cases the incongruent results, enhance the scientific necessity to better understand this technic.

A limitation of our study was the sample size, translated into a small subject number per group, which did not allow us to have better statistical strength. Objective methodologies to evaluate volunteer stress and anxiety should also be used, but unfortunately these tests were not included in our initial study methodology as we did not expected that a TMS based stimulation could cause this level of apparent student solicitude towards the procedure. Nevertheless, we tried to provide ideal protocol application conditions, previously by giving our volunteers all the information needed and during stimulation/recording procedures promoting a stress-free environment.

## Conclusions

Our results strongly support the hypothesis that TBS can effectively influence the cortical site of stimulation and also remote cerebral regions, directly or indirectly influencing neuronal excitatory/inhibitory networking, and that this influence is directly linked with stimulation characteristics and hemispheric lateralization. This significant capacity to modulate brain excitability should be further studied, either by neurophysiologic or behavioral testing in order to fully understand and dominate this noninvasive neuro-intervening tool. Further studies with larger subject number are required to confirm our findings and help understand whether these results have short duration, or if this neurocognitive influence is maintained for longer periods of time. We suggest also additional investigation studying and comparing these results using neuroimaging. It would be interesting to investigate the same protocol with repeated application of TBS in a daily scheme, with depression-like treatment sessions. Studies with a larger range of TBS intensities and different number of trains would also be important to evaluate in the future. We believe that P300 evoked potentials have the potential to be used as a useful tool to study and evaluate transcranial magnetic stimulation related outcomes.

## Supporting information

S1 File Data TBS_P300_PLOSONE. Study SPSS data.
(SAV)

S2 File Ethics Committee. (PDF)
