# Supplementary material for: Bilateral theta-burst magnetic stimulation influence on event-related brain potentials
Source: PLoS One. 2018 Jan 5;13(1):e0190693. doi: 10.1371/journal.pone.0190693 (PMC5755903; doi:10.1371/journal.pone.0190693)
Supplement: S2 File — (PDF) [file pone.0190693.s002.pdf]

UNIVERSIDADE DA BEIRA INTERIOR
FACULDADE DE CIÊNCIAS DA SAÚDE

PARECER

Processo: CE-FCS-2011-001

Data entrega processo: 30/08/2011

Data conclusão processo: 13/12/2011

Tema Projecto/Proponente: *"Estudo das respostas neuropsicológicas e neurofisiológicas cerebrais, em sujeitos normais, associadas à aplicação de campos magnéticos cerebrais"* – Profª. Doutora Maria Assunção Vaz Patto

Exma. Sra. Presidente da Faculdade de Ciências da Saúde

Apreciado o pedido referente ao processo acima mencionado esta Comissão não detectou matéria que ofenda os princípios éticos.

Covilhã, 11 Janeiro 2012

O Presidente da Comissão de Ética
Prof. Doutor José Martinez de Oliveira

O Vice-Presidente da Comissão de Ética
Prof. Doutor Joaquim Viana
